# Paired Box-1 (PAX1) Activates Multiple Phosphatases and Inhibits Kinase Cascades in Cervical Cancer

**DOI:** 10.1038/s41598-019-45477-5

**Published:** 2019-06-24

**Authors:** Po-Hsuan Su, Hung-Cheng Lai, Rui-Lan Huang, Lin-Yu Chen, Yu-Chi Wang, Tzu-I Wu, Michael W. Y. Chan, Chi-Chun Liao, Chien‐Wen Chen, Wei-Yu Lin, Cheng-Chang Chang

**Affiliations:** 1 Translational epigenetics center, Shuang Ho Hospital, Taipei Medical University, New Taipei City, Taiwan; 2 Department of Obstetrics and Gynecology, Shuang Ho Hospital, Taipei Medical University, New Taipei City, Taiwan; 3 Department of Obstetrics and Gynecology, School of Medicine, College of Medicine, Taipei Medical University, Taipei, Taiwan; 4 Graduate Institute of Life Sciences, National Defense Medical Center, Taipei, Taiwan; 5 Department of Obstetrics and Gynecology, Tri-Service General Hospital, National Defense Medical Center, Taipei, Taiwan; 6 Department of Obstetrics and Gynecology, Wan Fang Hospital, Taipei Medical University, Taipei, Taiwan; 7 Department of Life Science, National Chung Cheng University, Min-Hsiung, Chia-Yi, Taiwan; 8 Human Epigenomics Center, National Chung Cheng University, Min-Hsiung, Chia-Yi, Taiwan; 9 Northern Institute for Cancer Research, Newcastle University, Newcastle-upon-Tyne, UK

**Keywords:** Kinases, Cervical cancer, Tumour-suppressor proteins, Epigenomics, DNA methylation

## Abstract

DNA methylation alteration, such as global hypomethylation and localized hypermethylation, within the promoters of tumor suppressor genes, is an important risk factor in cervical cancer. The potential use of DNA methylation detection, in cervical cancer screening or triage of mildly abnormal cytology, has recently been demonstrated. In particular, *PAX1* DNA methylation testing was approved as an adjunct to cytology, in Taiwan, and is now undergoing registration trials in China. However, the function of *PAX1* in cancer biology remains largely unknown. Here, we show that PAX1 inhibits malignant phenotypes upon oncogenic stress. Specifically, PAX1 expression inhibited the phosphorylation of multiple kinases, after challenges with oncogenic growth factors such as EGF and IL-6. Analogously, PAX1 activated a panel of phosphatases, including DUSP1, 5, and 6, and inhibited EGF/MAPK signaling. PAX1 also interacted with SET1B, increasing histone H3K4 methylation and DNA demethylation of numerous phosphatase-encoding genes. Furthermore, hypermethylated *PAX1* associated with poor prognosis in cervical cancer. Taken together, this study reveals, for the first time, the functional relevance of PAX1 in cancer biology, and further supports the prospect of targeting multifold oncogenic kinase cascades, which jointly contribute to multiresistance, via epigenetic reactivation of *PAX1*.

## Introduction

Cervical cancer is the fourth-most common female cancer, and the fourth overall cause of cancer death, in the world. In 2018, there were an estimated 569,847 worldwide new cases, and 311,365 deaths^1^. Over 90% of deaths from cervical cancer occur in developing countries. However, even in developed countries, 13,240 and 62,356 annual new cases occur in the U.S and Southeast Asia, respectively^1,2^.

The causative agent of cervical cancer is the human papillomavirus (HPV)^3^. While most HPV infections are transient, few cells remain persistently infected. However, HPV persistent infection is insufficient for cancer formation, as the cervical epithelia must accumulate other genetic and epigenetic changes to become fully malignant^4^. To that end, genetic alterations, such as karotypic abnormalities (e.g., gains of chromosome 3q, 5p and 20q)^5–7^, amplification or activating mutations of oncogenes such as *PIK3CA* and *EGFR*, and inactivating mutations of tumor suppressor genes, such as *PTEN*, are all common contributors to cervical cancer^8–11^.

In addition to genetic events, epigenetic alterations, such as DNA methylation, play a critical role in cancer development and progression. A hallmark of cancer cells is a global decrease in DNA methylation, and localized hypermethylation of the promoters of specific tumor suppressor genes^12^. During the past decade, genome-wide approaches, such as microarray and next-generation sequencing (NGS), have more comprehensively identified differentially methylated genes in cancers. Moreover, several clinical trials are examining DNA methylation as a biomarker for cancer detection, including cervical and colon cancer^13,14^, with a methylation test for the advanced disease being approved in 2016 by the U.S. Food and Drug Administration (FDA)^15^.

Similar to the above, DNA methylation as a biomarker for cervical cancer early detection is also promising^16–18^. Cytology-based cervical cancer screening has been used for nearly a century, albeit with unsatisfactory sensitivity and a 20% false positive rate^19^. And, while a quadravalent vaccine against HPVs 5, 11, 16, and 18 has reduced infection by those strains, it is minimally effective against other strains, and is little available in developing nations^3^. To more accurately detect cervical cancer, DNA methylation, as an adjunct for cytology or human papilloma virus (HPV) testing, is becoming the standard for molecular cervical cancer screening^13,20^. The methylation of the paired box-1 (*PAX1*) gene in cervical cancer was firstly reported in 2008^16^. Serial validation studies performed worldwide have demonstrated its potential in cervical cancer molecular screening^21–24^. In 2016, the Taiwanese FDA approved the application of *PAX1* methylation as an adjunct of conventional cytology in cervical cancer screening^25^. Despite this diagnostic association with cervical cancer, however, there remains a gap in the mechanistic understanding of the precise role of *PAX1* in disease etiology.

The *PAX* gene family is named for the paired box DNA-binding domain that is critical for tissue development and cellular differentiation in embryos^26^. In cancer, however, the function of *PAX* genes is controversial and has not been well characterized^27^. Four PAX family subgroups exist, based on their structural domains: paired domain, octapeptide, and homeodomain^26^. *PAX1* and *PAX9* belong to subgroup I, which has a DNA-binding paired domain without a homeodomain, and these show functional redundancy during embryogenesis^28^. *PAX9* is also amplified and highly expressed in lung cancer, and its knockdown reduced lung cancer formation in a xenograft study, supporting a role in oncogenesis^29^. On the contrary, *PAX1* was aberrantly methylated, and downregulated, in cervical, oral, and ovarian cancer, suggesting that *PAX1* is a tumor suppressor gene^16,30,31^. Nevertheless, the mechanism underlying the role of the *PAX* gene family in cancer biology has not been elucidated. Here, we present the first comprehensive, functional investigation of the mechanistic role of *PAX1* in cancer.

## Results

### PAX1 Inhibits malignant phenotypes of cervical cancer cell lines upon EGF stimulation

**Figure 1:**
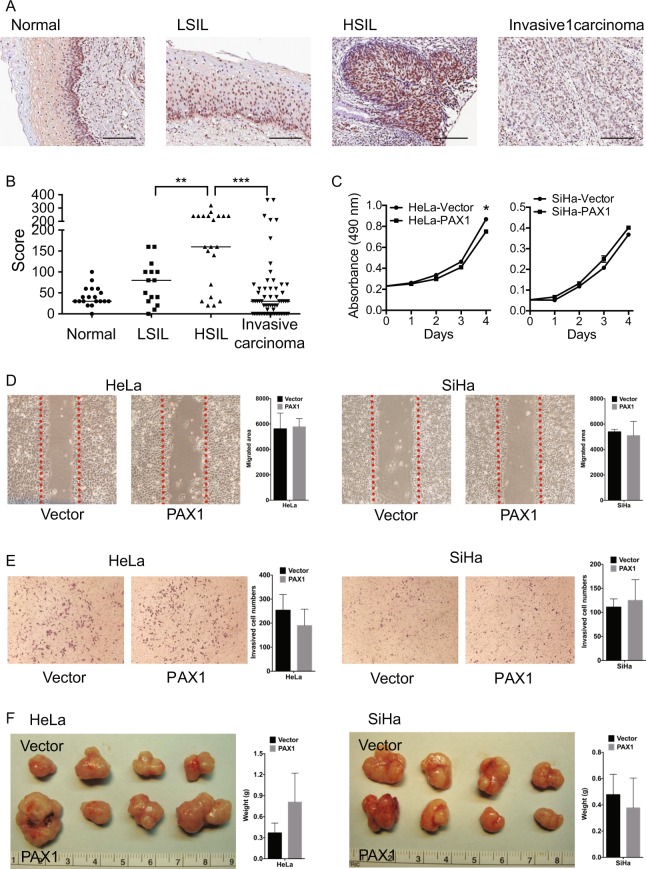
PAX1 slightly reduces malignant phenotypes in cervical cancer cell lines. (**A**) PAX1 protein expression in tissue samples across the full spectrum of cervical lesions. Normal, n = 19; LSIL, n = 15; HSIL, n = 21; and invasive carcinoma, n = 64. The scale bar represents 100 μm. The weak cytoplasmatic staining of PAX1 was background. (**B**) Scatterplot of PAX1 staining scores. **p < 0.01; ***p < 0.001. (**C**) *In vitro* growth curve of HeLa and SiHa cervical cancer cells in control and *PAX1*-overexpressing cells. Cancer cell migratory (**D**) and invasive (**E**) phenotypes, as assessed by wound healing and matrigel invasion assays, respectively. The red line in (**D**) indicates the migration start site. (**F**) *In vivo* tumor growth of HeLa and SiHa cells, in control and *PAX1*-overexpressing cells.

**Figure 2:**
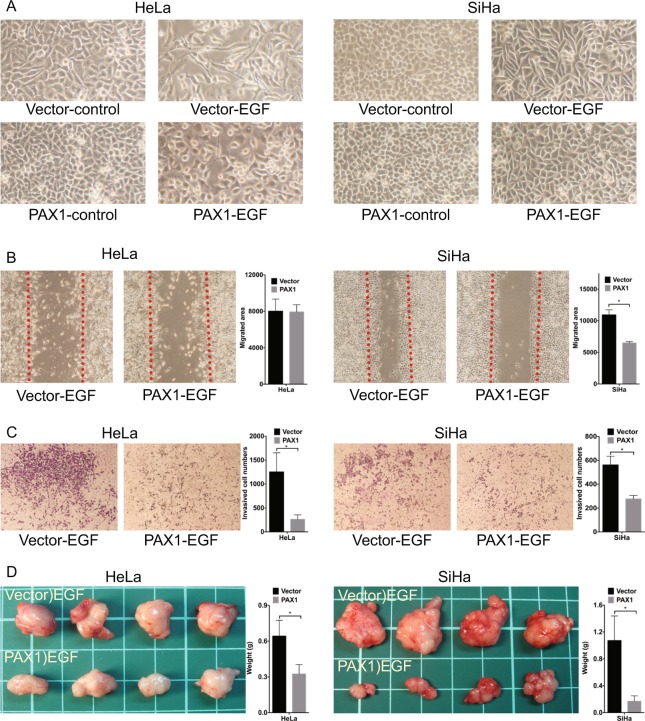
PAX1 inhibits EGF-induced malignant phenotypes in cervical cancer cell lines. (**A**) EGF-induced elongation and single-cell scattering of HeLa and SiHa cells is inhibited by PAX1, as are migratory (**B**) and invasive (**C**) phenotypes. The red line in (**B**) indicates the migration start site. (**D**) PAX1 inhibits tumor formation in HeLa and SiHa cell lines pretreated with EGF, *in vivo*.

To explore the role of PAX1 in cervical carcinogenesis, we used immunohistochemical staining to examine its expression over the full spectrum of cervical lesions, including normal epithelium, precancerous lesions, and invasive cancer. The results of this experiment revealed that PAX1 nuclear expression was weak in normal cervix, moderate in low-grade squamous intraepithelial lesion (LSIL), strongest in high-grade squamous intraepithelial lesion (HSIL), but weak in invasive cancer cells (Fig. 1A,B). These data suggest that PAX1 functions as a tumor suppressor, especially at the transition from *in situ* to invasive cancer. Consequently, we overexpressed *PAX1* in two cervical cancer cell lines, HeLa and SiHa, and found that it did not significantly inhibit malignant phenotypes, including proliferation, migration, and invasion, *in vitro*, and tumor growth, *in vivo* (Fig. 1C–F). However, the function of a tumor suppressor gene may not become obvious until the existence of oncogenic stressors, such as the expression of certain cytokines or growth factors. Thus, we hypothesized that PAX1 might inhibit malignant phenotypes in the presence of growth factors. In cervical cancer, the epidermal growth factor (EGF) was reported to induce the epithelial-to-mesenchymal transition (EMT), a metastasis-related phenotype that includes cancer cell invasion^32^. Hence, we examined the effects of PAX1 in the presence of EGF. As expected, PAX1 expression inhibited EGF-induced EMT phenotypes, including mesenchymal spindle shape, migration, and cell invasion (Fig. 2A–C). Analogously, we observed that the EMT marker Snail was downregulated by PAX1, while the epithelial marker CDH1 was upregulated (Supplementary Fig. S1). Moreover, PAX1 significantly inhibited tumor growth in cells pretreated with EGF (Fig. 2D). Together, these results support our hypothesis that PAX1 is a tumor suppressor that is responsive to environmental factors, such as EGF, and inhibits EGF-induced EMT and malignant phenotypes.

### PAX1 activates multiple phosphatases and inhibits EGF-mediated signaling

**Figure 3:**
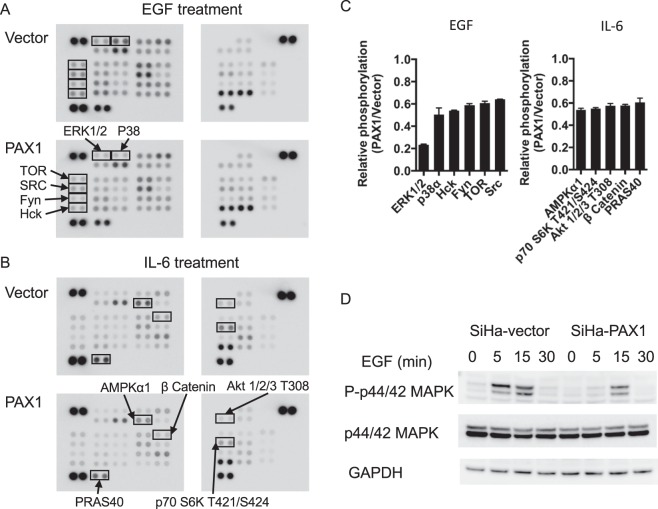
PAX1 inhibits the EGF- and IL-6 mediated kinome. Kinase array revealing PAX1 inhibition of EGF- (**A**) and IL-6- (**B**) kinase activity, and signaling, in HeLa cells. (**C**) Quantitative results of PAX1-inhibited pathway signaling. (**D**) Western blot confirming decreased ERK1/2 phosphorylation by PAX1, in SiHa cells. GAPDH was used as loading control. Full blots are shown in Supplementary Fig. S3.

To examine the extent to which PAX1 inhibits the EGF-associated kinome, we tested for kinome changes using an array-based assay. The results of this experiment demonstrated that PAX1 inhibited the activity of multiple oncogenic kinases, including the MAPK pathway (ERK1/2 and P38), and the SRC pathway (HCK, FYN, and SRC) (Fig. 3A,C, and Supplementary Fig. S2A). In addition to EGF, IL-6 is another oncogenic cytokine, and its activation can induce STAT3 and AKT signaling, resulting in tumor growth and invasion in numerous cancers, including cervical^33,34^. Furthermore, IL-6 expression has been correlated with poor prognosis in cervical cancer^35^. In this context, we observed PAX1 to inhibit IL6-mediated activation of several oncogenic signaling kinases, such as AMPK, P70 S6 kinase, AKT, β-catenin, and PRAS40 (Fig. 3B,C and Supplementary Fig. S2B).

ERK1/2 is most sensitive to PAX1 inhibiting its phosphorylation by about 80%. Western blotting thus confirmed that PAX1 delayed the timing and amplitude of ERK1/2 phosphorylation in SiHa cells (Fig. 3D). Since inhibition of EGF signaling may be achieved via activation of protein phosphatases, and a previous study revealed the protein tyrosine phosphatase, receptor type R (PTPRR), to inhibit ERK phosphorylation in cervical cancer^17^, we tested whether PTPRR is a transcriptional target of PAX1. Those results demonstrated that PTPRR was indeed upregulated upon *PAX1* expression, especially in the presence of EGF (Supplementary Fig. S4A). Further, ChIP-PCR confirmed PAX1 binding to the *PTPRR* promoter (Supplementary Fig. S4B). These results reveal that PAX1-activated PTPRR inhibits the EGF signaling pathway, via inhibition of ERK1/2 phosphorylation.

**Figure 4:**
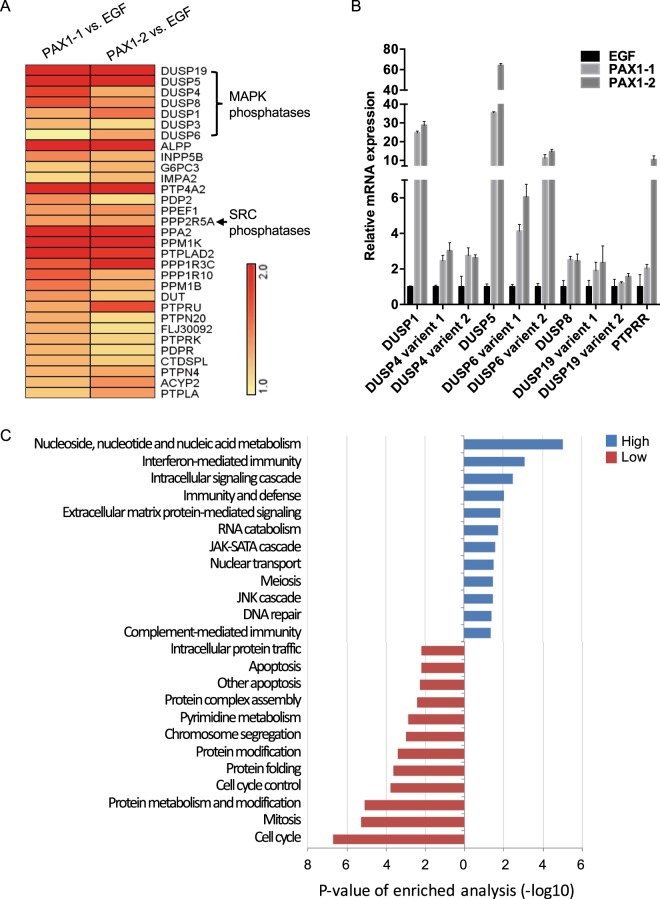
Genome-wide analysis reveals that PAX1 induces multiple phosphatases upon EGF stimulation. (**A**) Heatmap of PAX1-induced phosphatases in HeLa cell. (**B**) Validation of phosphatase expression by qPCR. (**C**) Gene ontology analysis of PAX1-regulated genes.

As PAX1 can also inhibit multiple kinase pathways, we assessed this possibility, in a manner similar to our observations for PTPRR. Our analysis of gene expression profiles obtained after *PAX1* expression and EGF stimulation (Supplementary Fig. S5A), demonstrating that 2534 genes were upregulated by PAX1, including 32 phosphatase genes (Supplementary Fig. S5B, Fig. 4A and Supplementary Table S1). Of these, six dual-specificity phosphatases (DUSPs; DUSP1, DUSP4, DUSP5, DUSP6, DUSP8, and DUSP19) are known to dephosphorylate ERK^36^, and PPP2R5A, a subunit of PP2A, is known to dephosphorylate SRC^37^. The expression of these six DUSPs was confirmed by qPCR, among which DUSP5, DUSP1, and DUSP6 were dramatically upregulated by 60-, 50-, and 15-fold, respectively (Fig. 4B), and PAX1 was confirmed to bind the *DUSP1*, *DUSP5*, and *DUSP6* promoters (Supplementary Fig. S6). Gene ontology analysis also revealed that PAX1 reduced the genes for cell-cycle and mitosis pathways (Fig. 4C and Supplementary Table 2), further indicative of it tumor suppressor role.

### A PAX1–WDR5–SET1B complex methylates H3K4 and activates the expression of multiple phosphatases

**Figure 5:**
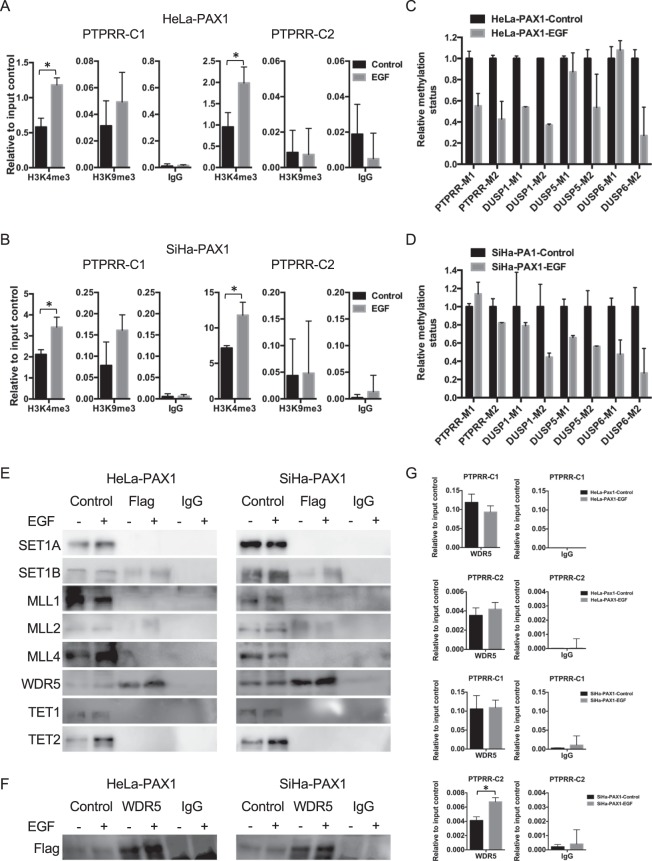
PAX1 regulates phosphatases’ expression by recruiting the histone methyltransferase SET1B. The status of histone H3K4 and H3K9 trimethylation within the *PTPRR* promoter in *PAX1*-expressing cells after EGF treatment. (**A**) HeLa- and (**B**) SiHa-*PAX1*-expressing cells. (**C**,**D**) Promoter methylation status of phosphatase genes, in *PAX1*-expressing cells, after EGF treatment. Interaction between PAX1, SET1B, histone H3K4 HMT, and DNA demethylases, as detected by co-IP. An anti-FLAG antibody was used for immunoprecipitation, and antibodies against the histone H3K4 HMT complex and DNA demethylases were used for blotting (**E**). The reverse experiment, i.e., complexes immunoprecipitated by an antibody against WDR5, and immunoblot for Flag (**F**). (**G**) The binding of WDR5, within the *PTPRR* promoter in PAX1-expressing cells, after EGF treatment. Full blots are shown in Supplementary Figs S8 and S9.

To clarify the mechanism of PAX1 activation of DUSPs, we assessed changes in histone modifications in various *DUSP* genes. ChIP-PCR showed that PAX1 expression increased trimethylation of histone H3, lysine 4 (H3K4, an activating modification), without changing H3K9 trimethylation (a repressive modification), within the *PTPRR*, *DUSP1*, *DUSP5*, and *DUSP6* promoters (Fig. 5A–B and Supplementary Fig. S7). Moreover, we observed reduced levels of DNA methylation within these same promoters (Fig. 5C,D). These results suggest that PAX1 cooperates with H3K4 methyltransferases and/or H3K9/DNA demethylases, to derepress phosphatase expression.

Two major enzyme families are involved in H3K4 trimethylation in humans: the SET and MLL families. Both share common components, such as WDR5, but target diverse genes^38^. To understand how PAX1 might induce H3K4 trimethylation, we tested possible protein-to-protein interactions between PAX1 and components of the SET and MLL complexes, including SET1A, SET1B, MLL1, MLL2, MLL4, and WDR5, in addition to the DNA demethylases, TET1 and TET2. Coimmunoprecipitation assays indicated direct binding of PAX1 to SET1B and WDR5, but not to other H3K4 HMTs and DNA demethylases (Fig. 5E). Analogously, the reverse experiment also demonstrated WDR5 binding to PAX1 (Fig. 5F), which also bound the *PTPRR*, *DUSP1*, *DUSP5*, and *DUSP6* promoters (Fig. 5G and Supplementary Fig. S6). Taken together, these data suggest that PAX1 activates phosphatase genes, via interacting with SET1B and WDR5, to change histone modifications to a transcriptionally permissive state.

### PAX1 methylation associates with poor prognosis in cervical cancer

**Figure 6:**
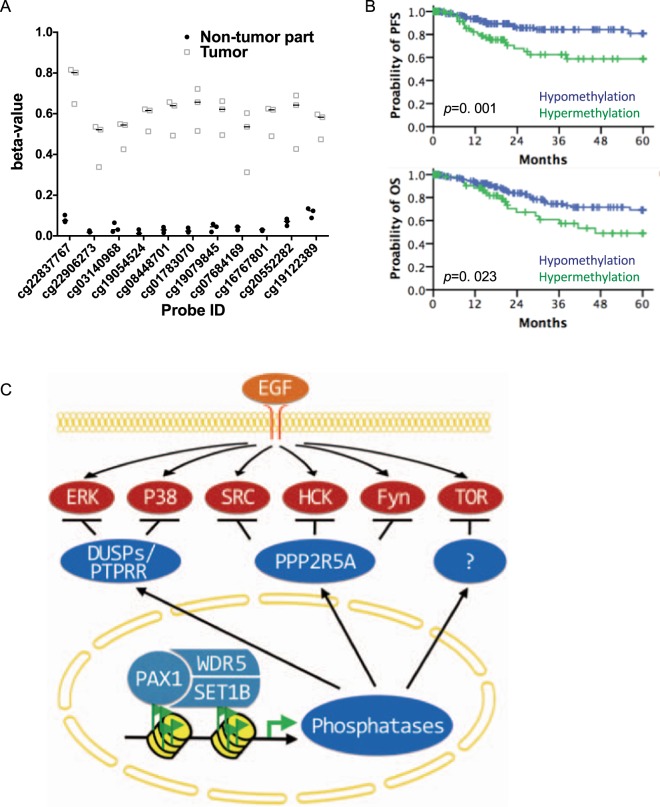
*PAX1* promoter methylation status in cervical cancer, and association with decreased progression-free (PFS) and overall (OS) survival. (**A**) Methylation status of *PAX1* in cervical cancer. Displayed are the methylation levels of three patients, who donated both tumor and non-tumor specimens. T, tumor tissue; N, normal tissue. The analyzed probes were located in the CpG island (chr20:21686200-21687689) of PAX1 promoter. (**B**) Kaplan–Meier plots of the probability of PFS and OS, stratified according to the methylation status of *PAX1*, in patients with cervical cancer. (**C**) Proposed model of PAX1-induced phosphatase expression, and disruption of EGF signaling.

To understand further the status of *PAX1* promoter methylation in cervical cancers, we extracted and analyzed DNA methylation profiles from a TCGA dataset. The median methylation levels (beta-values) of the non-tumor specimens and tumors were 0.05 and 0.58, respectively (Fig. 6A), while *PAX1* promoter methylation levels in non-tumor tissues were significantly lower than those in tumors (Mann-Whitney U test, *p* < 0.0001). In addition, hypermethylated *PAX1* associated with poor progression-free survival (PFS), and poor overall survival (OS), in patients with cervical squamous cell carcinoma (Fig. 6B).

## Discussion

The present study demonstrated that PAX1 plays a tumor suppressor role in response to the onset of oncogenic stress, including growth factor stimulation. We further demonstrated that PAX1 inhibited EGF and IL-6 signaling, through epigenetic activation of multiple phosphatases (Fig. 6C). Kinase–phosphatase network is rigidly regulated in normal physiology^39–41^. The kinase cascade may reciprocally activate the phosphatase to keep growth signaling in check. In cancer, however, the regulation loop between kinases and phosphatases is little known. Our data show that PAX1 inhibits EGF–ERK signaling, via epigenetic activation of multiple phosphatases. Furthermore, it was reported the ERK1 mediates activation of the PAX1-like protein in *Giardia lamblia*^42^, suggesting that PAX1 mediates a physiologic balance between kinases and phosphatases. Taken together, we speculated that PAX1 might activate multiple phosphatases that keep oncogenic kinases in check.

Our previous study demonstrated that the methylation of *PAX1* is associated with HPV L1 region methylation in the ≥CIN2 lesions^25^. Under nitroxidative stress, full-length HPV transformed precancerous cell lines can undergo epigenomic modifications including DNA methylation of an EMT inhibition phosphatase, *PTPRR*^43^. Indeed, there were reports demonstrating HPV infection is associated with higher nitric oxide synthase expression^44^ and nitric oxide (NO) concentration in cervix^45^, suggesting the indirect effects of HPV infection on epigenomics. Furthermore, the methylation of *PAX1* was increased in the keratinocyte after immortalized by the full-length HPV16^46^. Together with the present study, the infection of HPV may cause the promoter methylation of *PAX1* and result in the reduction of phosphatases’ expression. Therefore, hypermethylated *PAX1* may derepress the oncogenic kinase cascade, thus promoting cancer progression.

Previously, the PAX family was reported to participate in development, but its role in cancer has been little studied. It was suggested that PAX2 and PAX5 play tumor suppressor roles in ovarian cancer and leukemia^47,48^, while conversely, PAX3, PAX7, and PAX6 were reported to be oncogenic in embryonal rhabdomyosarcomas and retinoblastoma^49,50^. However, these reports were all based only on correlations between expression and malignant phenotypes, and the underlying mechanisms were not investigated. Although PAX1 is known to participate in neuronal development^51^, there have been no reports of a possible role in cancer biology. Previous studies regarding the PAX family and chromatin modifiers (histone deacetylases (HDACs) and histone lysine methyltransferases (HMTs)), in developmental biology, revealed interactions between PAX2 and MLL2/3 (KMT2C/D, an HMT), in embryonic kidney cell lines, PAX5 and HMTs/HDACs, in mouse B cell development, and PAX7 and HMTs, in mouse myogenesis^52–55^. In cancer, PAX-interacting proteins remain largely unknown. Here, we demonstrate that PAX1 interacts with SET1B, but neither MLL1–MLL4 nor SET1A. Further, the cooperation between PAX1 and the SET1B histone H3K4 methyltransferase complex c activated the phosphatome and suppressed kinase signaling. These findings represent the first indication of PAX1-modulated epigenetic regulation of the kinase–phosphatase loop in cancer biology.

In addition, resistance to targeted therapy in cancer is a notorious problem. For example, tyrosine kinase inhibitors (TKIs), designed to target mutant EGFR, have shown dramatic therapeutic efficacy in lung cancer; however, patients often develop resistance to therapy^56^. Additionally, compensatory oncogenic kinase cascades, such as the MAPK^57^, PI3K/AKT^58^, and SRC/AKT^59^ pathways, may render TKIs ineffective. In that regard, our study revealed that PAX1-activated phosphatases can inhibit multiple MAPK pathways and the SRC kinase. Consequently, reactivation of DNA methylation-silenced *PAX1* may be a strategy to inhibit multiple oncogenic kinase pathways, which could reverse multi-pathway resistance to antineoplastic TKI drugs.

In conclusion, PAX1 is responsible for maintaining an on-and-off homeostasis between kinases and phosphatases, within the cervical epithelium. Loss of *PAX1* expression, however, through DNA methylation, may disrupt this balance, leading to cancer development, supporting the further study of *PAX1* DNA methylation as a biomarker for cervical cancer detection. Moreover, reactivation of PAX1 may overcome the failure of current target therapies targeting single kinases in cervical cancer treatment. We strongly contend that such epigenetic therapies, and consequent reactivation of tumor suppressors, are a promising strategy for overcoming the insidious problem of cancer chemo- and targeted therapy multiresistance.

## Methods

### Clinical samples

Paraffin-embedded cervical tissues of Chinese patients were retrieved from the Department of Pathology, Tri-Service General Hospital, National Defense Medical Center, Taiwan. The tissue microarrays comprised histologically normal squamous epithelium (n = 19), mild dysplasia (n = 15), carcinoma *in situ* (n = 21) and invasive carcinoma (n = 46). A standard protocol for immunohistochemistry was used with rabbit polyclonal anti-human PAX1 antibody (Abcam, ab95227). All tissue microarray slides were examined and scored by pathologists, according to the summation of the percentage of area stained multiplied by the stain intensity. All patients were diagnosed, treated, and had their tissues banked at the Tri-Service General Hospital, National Defense Medical Center, Taipei, Taiwan. The final diagnosis was made by tissue-proven pathology rather than cytology except for controls. Exclusion criteria included pregnancy, chronic or acute systemic viral infections (except HPV infection), a history of cervical neoplasia, skin or genital warts, an immunocompromised state, the presence of other cancers, or a history of surgery to the uterine cervix. This study was conducted in accordance with the Declaration of Helsinki and approved by the Institutional Review Board of the Tri-Service General Hospital. All of the patients signed informed consent forms before study.

### Cell lines, culture conditions, and constructs

The human cervical cancer cell lines HeLa and SiHa were cultured in Dulbecco’s modified Eagle’s medium (DMEM) or RPMI 1640 medium containing 10% (w/v) fetal calf serum, penicillin at 100 U/ml, streptomycin at 100 μg/ml, and L-glutamine at 2 mmol/l (all from Thermo Fisher Scientific). PAX1 (NM_006192) was constructed by inserting a full-length cDNA product into a pCMV-Tag 2B vector. The construct was then transfected into HeLa and SiHa cells with Lipofectamine 2000 (Thermo Fisher Scientific) in Opti-MEM I reduced-serum medium (Thermo Fisher Scientific) at 37 °C in a 5% CO_2_ atmosphere for 4–5 h, after which the medium was removed and replaced with fresh culture medium. Cells were selected by antibiotic (G418) after 2 days in culture. Control cells were transfected with pCMV-Tag 2B vector only. The concentration of EGF (Thermo Fisher Scientific) in culture medium in this study is 50 ng/mL.

### Cell proliferation assay

Cell proliferation was measured using MTS assay (Promega) as described in our previous study^43^. Briefly, cells were seeded in 96-well plates at a density of 2000 cells/well. On days 0, 1, 2, 3, and 4, culture medium was replaced by fresh medium containing MTS reagent (100 µl medium plus 20 µl MTS reagent/well). After incubation at 37 °C for 2 hours, the absorbance at 490 nm was measured. Experiments were repeated four times.

### Cell migration assay and invasion assays

Cell migration assay and invasion assays were described in detail in our earlier study^43^. Cell migration was assessed using the scratch wound-healing assay. Cells were seeded into 6-well dishes until confluent and then wounded using a pipette tip. The migration area was photographed at 0 and 24 h. Cell migration was quantified by measuring the migrated area using ImageJ. Cell invasion was measured in the Transwell system with Matrigel (BD Bioscience). 2000 cells were seeded in the upper chamber in culture medium without serum. The same medium with serum was added to the lower chamber. Cells that had permeated the Matrigel and migrated to the bottom of the insert were fixed with methanol and stained with Giemsa’s azur eosin methylene blue solution (Merck) after 24 h. The cells in each chamber were photographed and counted.

### RNA extraction, cDNA synthesis, and quantitative real-time PCR (qPCR)

RNA extraction, cDNA synthesis, and quantitative real-time PCR were performed as described in our earlier studies^17,43^. Total RNA was isolated by Qiagen RNeasy kit with DNase I digestion (Qiagen) and reverse-transcribed to cDNA using the Super Script III first-strand synthesis system for RT-PCR with Oligo(dT) as the primer (Thermo Fisher Scientific). qPCR was performed by Roche SYBR Green Real-Time PCR System (Roche). The glyceraldehyde-3-phosphate dehydrogenase (*GAPDH*) was used as the internal reference gene. The comparative threshold cycles (Ct) method was used for relative quantification. All values are expressed as mean ± SD. The primers used in this study are shown in Supplementary Table S3.

### DNA extraction, bisulfite modification, quantitative methylation-specific PCR (qMS-PCR)

qMS-PCR was performed as described by in our former study^43^. In brief, genomic DNA was extracted with the QIAmp DNA Mini Kit (Qiagen) and bisulfite-modified by CpGenome Fast DNA Modification Kit (Millipore). qMS-PCR was performed using SYBR Green Real-Time PCR System (Roche). Internal control gene was the type II collagen (*COL2A*). Samples with a crossing point-PCR-cycle (Cp) value for *COL2A* greater than 36 were defined as failure to detect. The DNA methylation level was assessed as the methylation index (M-index), 10 000 × 2([Cp of *COL2A*] − [Cp of gene]. The primers used in this study are shown in Supplementary Table S4.

### Chromatin immunoprecipitation assay

Chromatin immunoprecipitation (ChIP) assays were performed according to the protocol from Millipore (EZ-Magna ChIP G Chromatin Immunoprecipitation Kit). DNA was eluted with 100 ul elution buffer, and 4 ul was used per Q-PCR analysis. Quantitative PCR was performed using RT² SYBR Green qPCR Master Mixes (Qiagen) in a LightCycler 480 Real-Time PCR System (Roche). The antibodies used in the ChIP-PCR analysis were anti-Flag (Sigma, F1804); anti-trimethyl-Histone H3K4 (07-473), anti-trimethyl-Histone H3K9 (07-442) and anti-mouse IgG (12-371) were from Merck Millipore. The primers used in this study are shown in Supplementary Table S5.

### Immunoblot and kinase array analysis

Cells used for immunoblot were serum-starvated for 16 h and treated with medium with EGF for 0, 5, 15 and 30 min. The process of immunoblot were described in detail in our earlier study^43^. The antibodies used in the immunoblot analysis were: anti-phospho-ERK 1/2 (9106), anti-ERK 1/2 (9102) from Cell Signaling; anti-Set1A (sc-515590), anti-Set1B (sc-248564), anti-MLL1 (sc-377274), anti-MLL2 (sc-292359), anti-MLL4 (sc-517017), anti-TET2 (sc-136926), anti-TET3 (sc-139186) from Santa Cruz; anti-TET1 (GT1462) from GeneTex. Kinome was analyzed by Human Phospho-Kinase Array Kit (ARY003B, R & D systems) according to the manufacturer’s instructions. Cells used for kinome analysis were serum starvated for 16 h and treated with medium with EGF or IL-6 for 15 min.

### *In vivo* tumorigenicity model

Six-week-old nude mice were used in the tumorigenicity analysis. 10^6^ cells from each stable line were resuspended in 0.1 ml PBS and injected subcutaneously into both flanks of each mouse. The mice were sacrificed at day 30. Tumors were removed from the mice and weighed. The protocol for this animal experiment was approved by the Institutional Animal Care and Use Committee (IACUC) of the National Defense Medical Centre, Taipei, Taiwan. All animal procedures and animal care were performed according to institutional animal research guidelines.

### Global gene expression analysis

Total RNA samples were isolated and the absorbance ratio of A260/A280 and A260/A230 analyzed by Nanodrop 2000 (Thermo Scientific) should bigger than 1.8 and 1.0, respectively. The further qualification, the ratio of 28S/18S and RNA quality indicator (RQI) was analyzed by Agilent 2100 Bioanalyzer (Agilent Technologies). Samples with ratio of 28S/18S is bigger than 1 and RQI is bigger than 7 were send to the core service unit (Health GeneTech Corp.) for whole-genome expression analysis. We measured gene expression profiles using Illumina HumanHT-12 v4 Expression Beadchip (Illumina, Inc.). The data had been deposited in the Gene Expression Omnibus (GEO) database (GSE102986). After quantile normalization, we removed the probes with detecting *p*-values > 0.05 in all of the samples. The average of signal (AVG_signal) of each probe was calculated from duplication of expression data. Differentially expressed genes were identified as having a ≥1.25-fold change in the average expression in the PAX1-1 set and PAX1-2 set compare to EGF set (Supplementary Fig. S5A,B). The phosphatase gene list was obtained from the human DEPhOsphorylation database (DEPOD, http://depod.bioss.uni-freiburg.de/).

### Statistical analysis

The Mann-Whitney U test, two-tailed, was used to compare data groups for cell proliferation, migration, invasion, and tumor formation. Standard deviations were used for error bars and various comparisons. Kaplan-Meier analysis and log-rank tests were used to calculate survival, and to compare differences between curves for progression-free survival (PFS) and overall survival (OS). *p-*values < 0.05 were considered to be statistically significant. Methylomics profiles of cervical cancer cases, obtained from The Cancer Genome Atlas (TCGA), were based on data generated using Human Methylation 450 BeadChips (Illumina). The results were downloaded from the Broad Institute GDAC Firehose (http://gdac.broadinstitute.org/), and used in compliance with TCGA’s data usage policy.

## Supplementary information

Supplemental data

Supplemental Table S2

## Data Availability

All data generated or analysed during this study are included in this published article and its supplementary information files. The whole-genome expression data had been deposited in the Gene Expression Omnibus (GEO) database (GSE102986).
