# Supplementary material for: Paired Box-1 (PAX1) Activates Multiple Phosphatases and Inhibits Kinase Cascades in Cervical Cancer
Source: Sci Rep. 2019 Jun 24;9:9195. doi: 10.1038/s41598-019-45477-5 (PMC6591413; doi:10.1038/s41598-019-45477-5)
Supplement: Supplementary file 1 — Supplemental data [file 41598_2019_45477_MOESM1_ESM.pdf]

Supplementary:

Paired Box-1 (PAX1) Activates Multiple Phosphatases and Inhibits Kinase Cascades in Cervical Cancer

Running title; PAX1 as a tumor suppressor in cervical cancer

Po-Hsuan Su[1, 2,†], Hung-Cheng Lai[1, 2, 3,†], Rui-Lan Huang[2, 3], Lin-Yu Chen[4], , Yu-Chi Wang[5], Tzu-I Wu[3, 6], Michael WY Chan[7, 8], Chi-Chun Liao[2], Chien-Wen Chen[2], Wei-Yu Lin[9], Cheng-Chang Chang[5, *]

†These authors contributed equally to this work.

The authors declare no potential conflicts of interest.

1. Translational epigenetics center, Shuang Ho Hospital, Taipei Medical University, New Taipei City, Taiwan.
2. Department of Obstetrics and Gynecology, Shuang Ho Hospital, Taipei Medical University, New Taipei City, Taiwan.
3. Department of Obstetrics and Gynecology, School of Medicine, College of Medicine, Taipei Medical University, Taipei, Taiwan.
4. Graduate Institute of Life Sciences, National Defense Medical Center, Taipei, Taiwan.
5. Department of Obstetrics and Gynecology, Tri-Service General Hospital, National Defense Medical Center, Taipei, Taiwan.
6. Department of Obstetrics and Gynecology, Wan Fang Hospital, Taipei Medical University, Taipei, Taiwan.

9. Northern Institute for Cancer Research, Newcastle University, Newcastle-upon-Tyne, UK.

*Correspondence author:

Hung-Cheng Lai, M.D., Ph.D.,

No.291, Jhongjheng Rd., Jhonghe , New Taipei 23561, Taiwan

E-mail: hclai30656@gmail.com; hclai@s.tmu.edu.tw.

Supplemental Figure 1

A

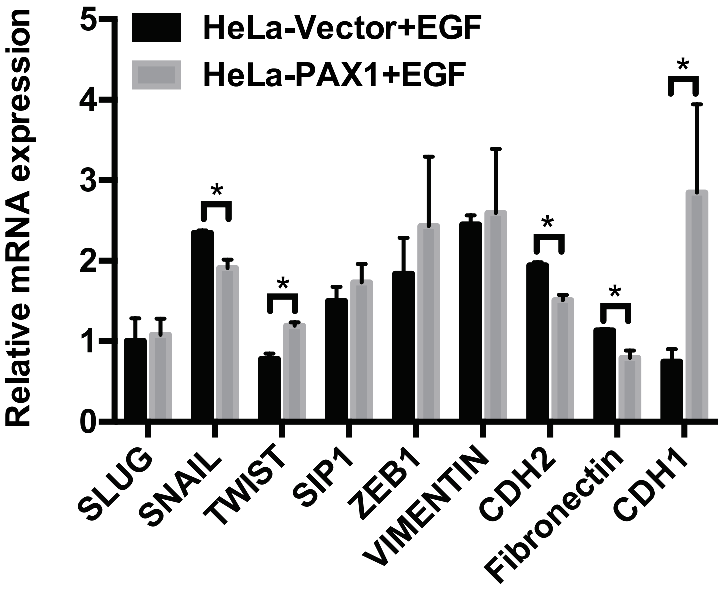

B

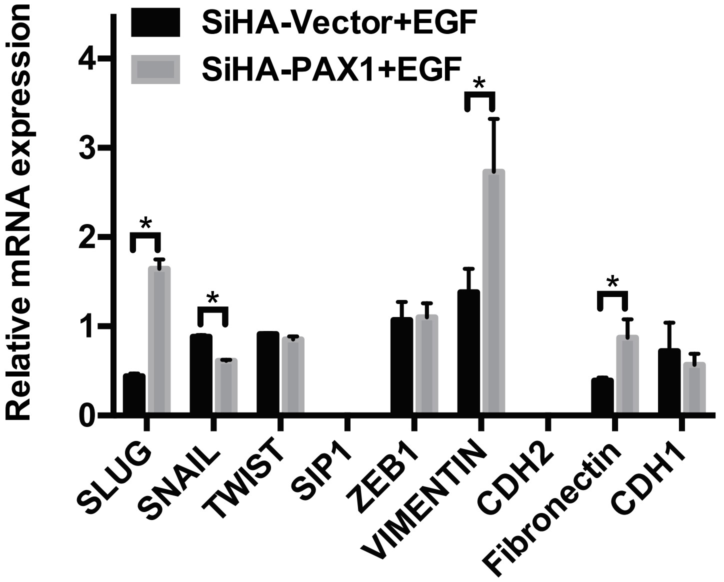

Supplemental Figure 2

A

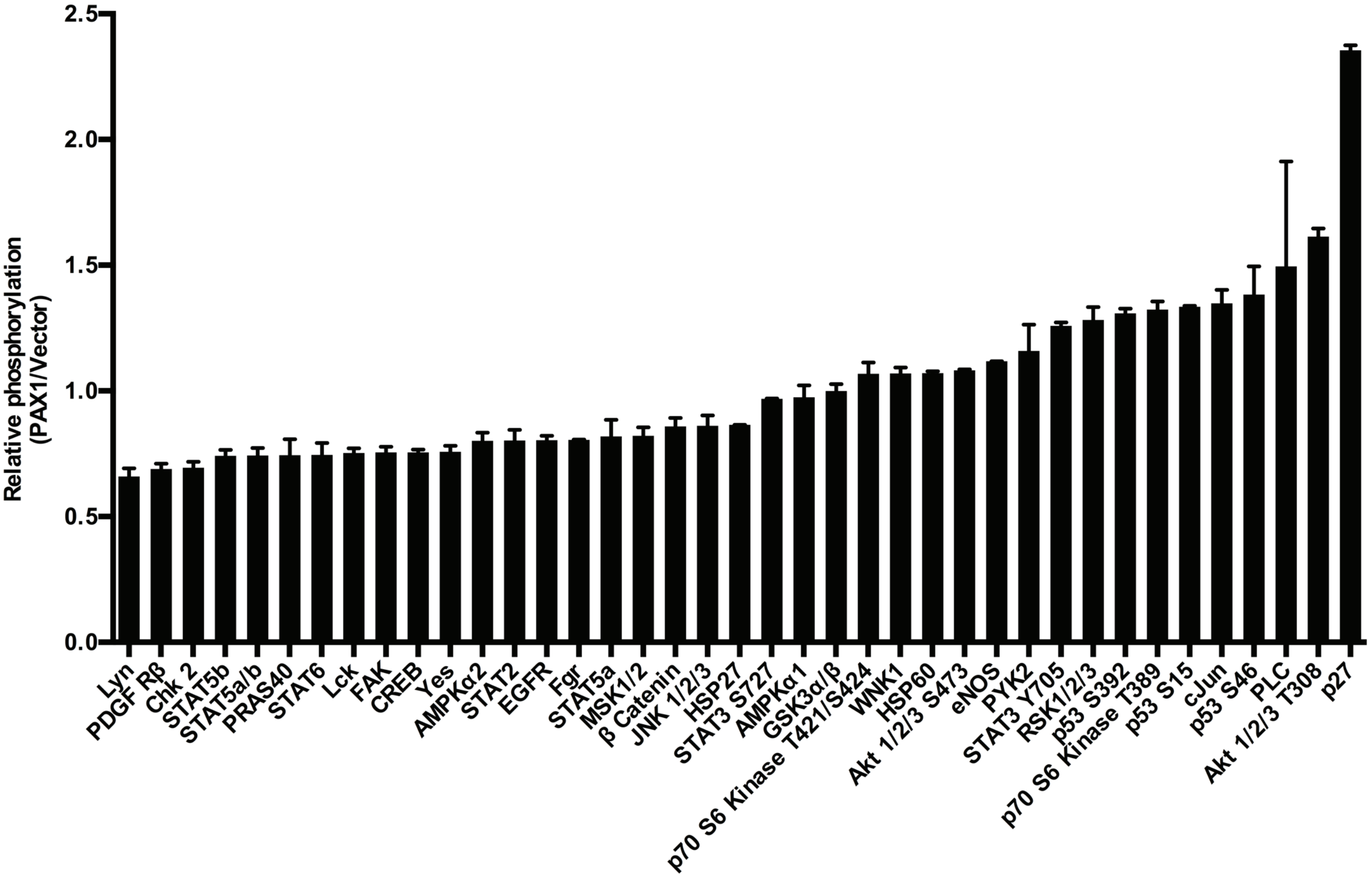

B

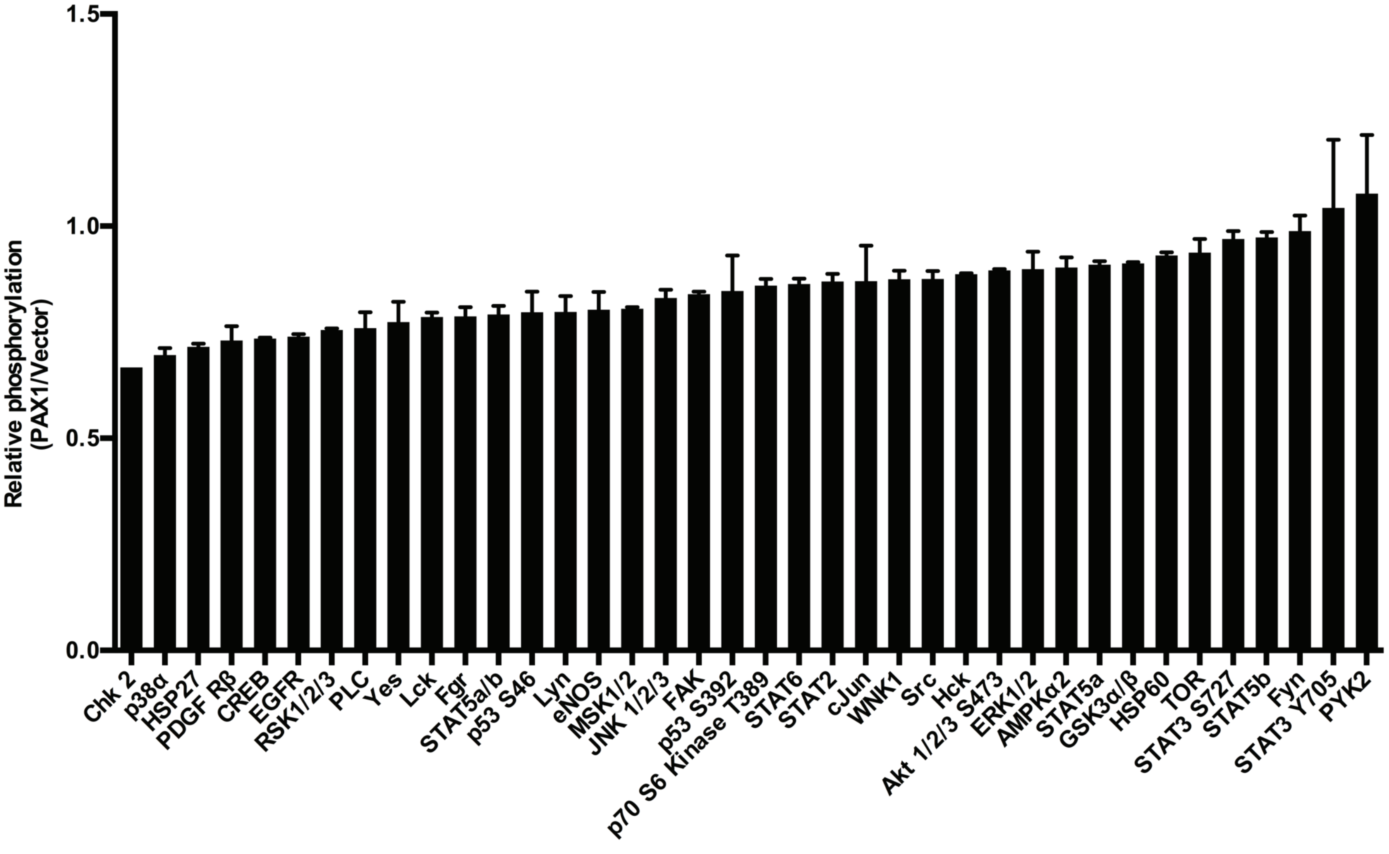

Supplemental Figure 3

A

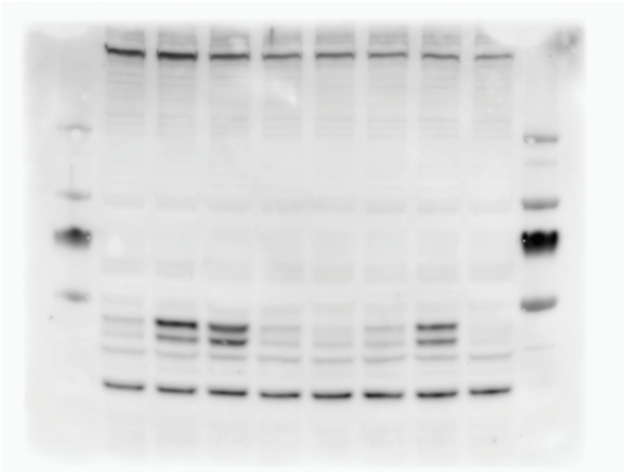

B

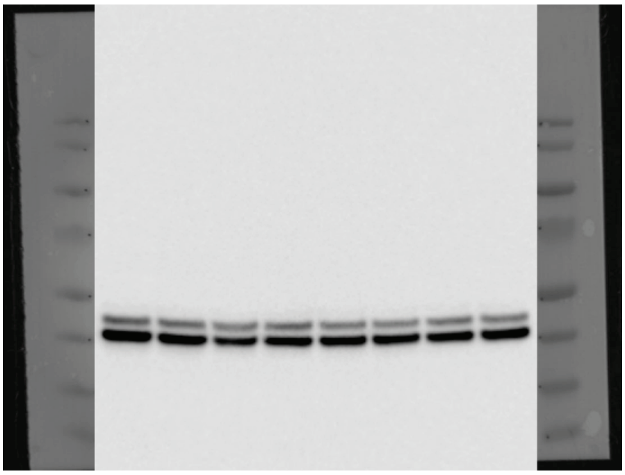

Supplemental Figure 4

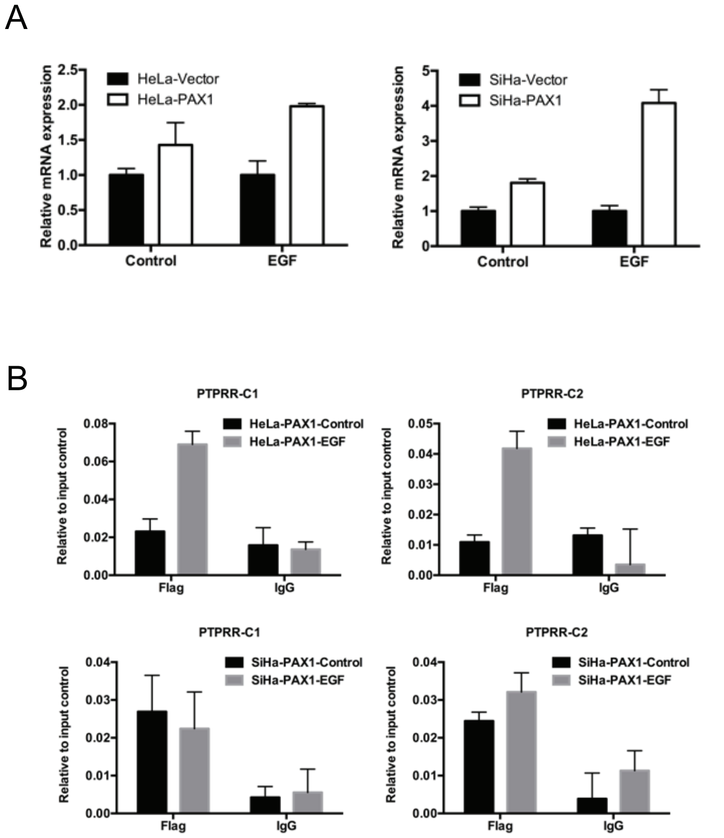

Supplemental Figure 5

A

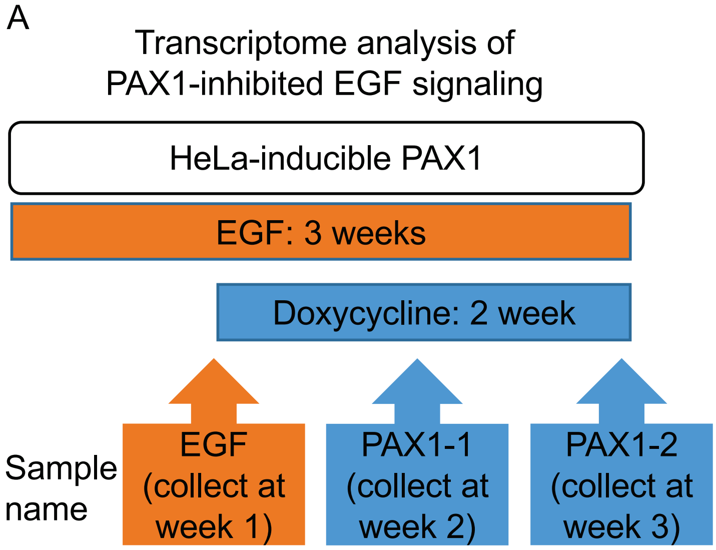

B

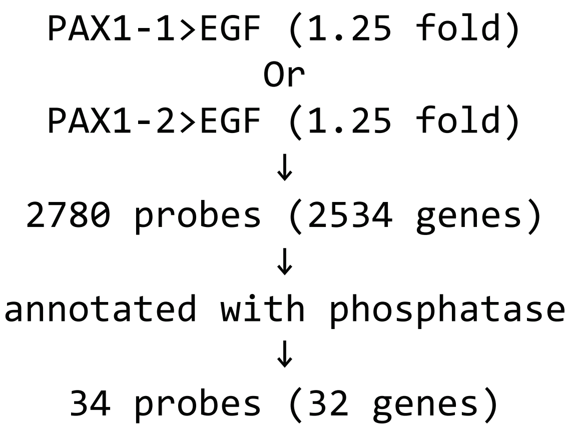

Supplemental Figure 6

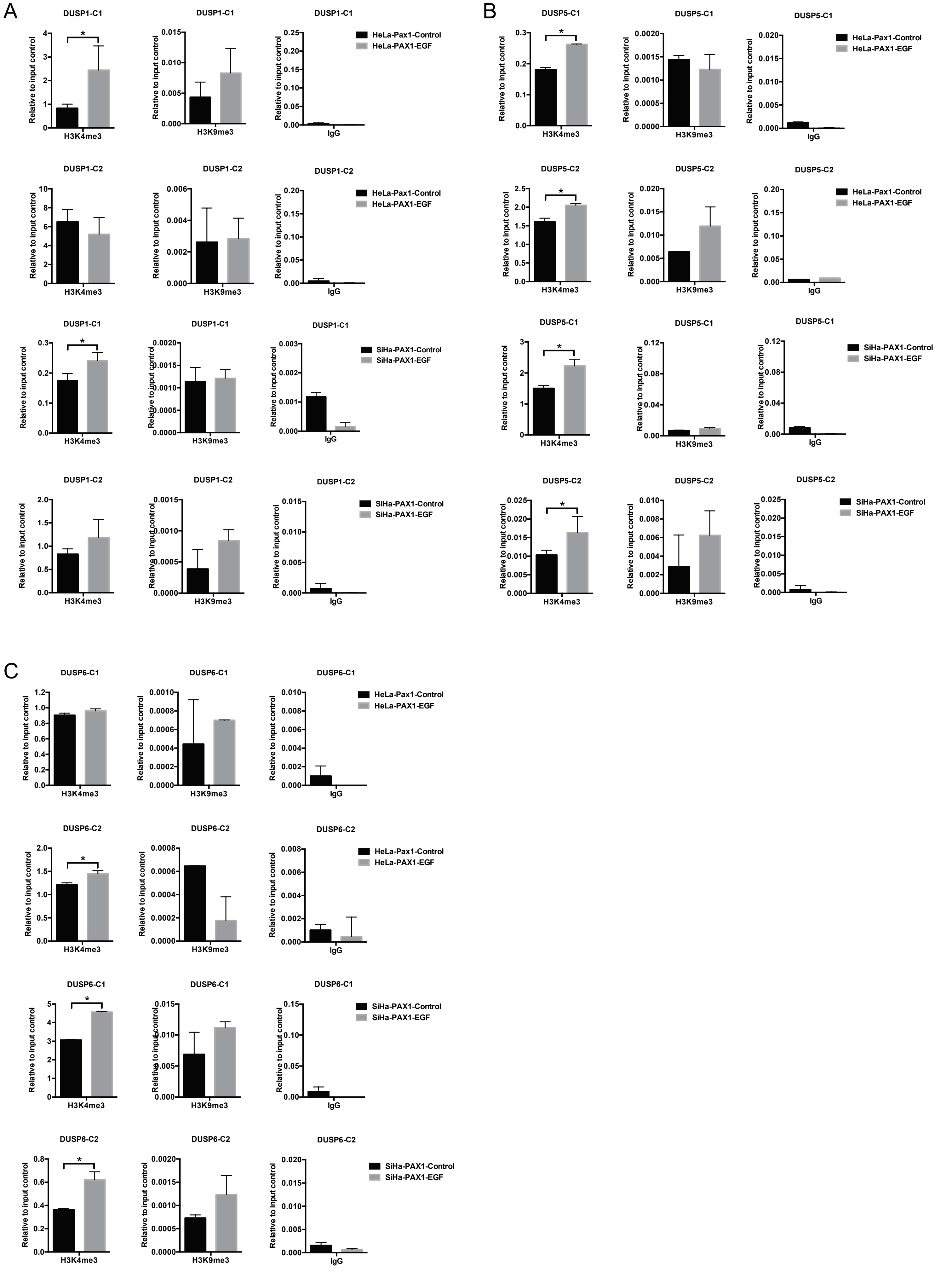

Supplemental Figure 7

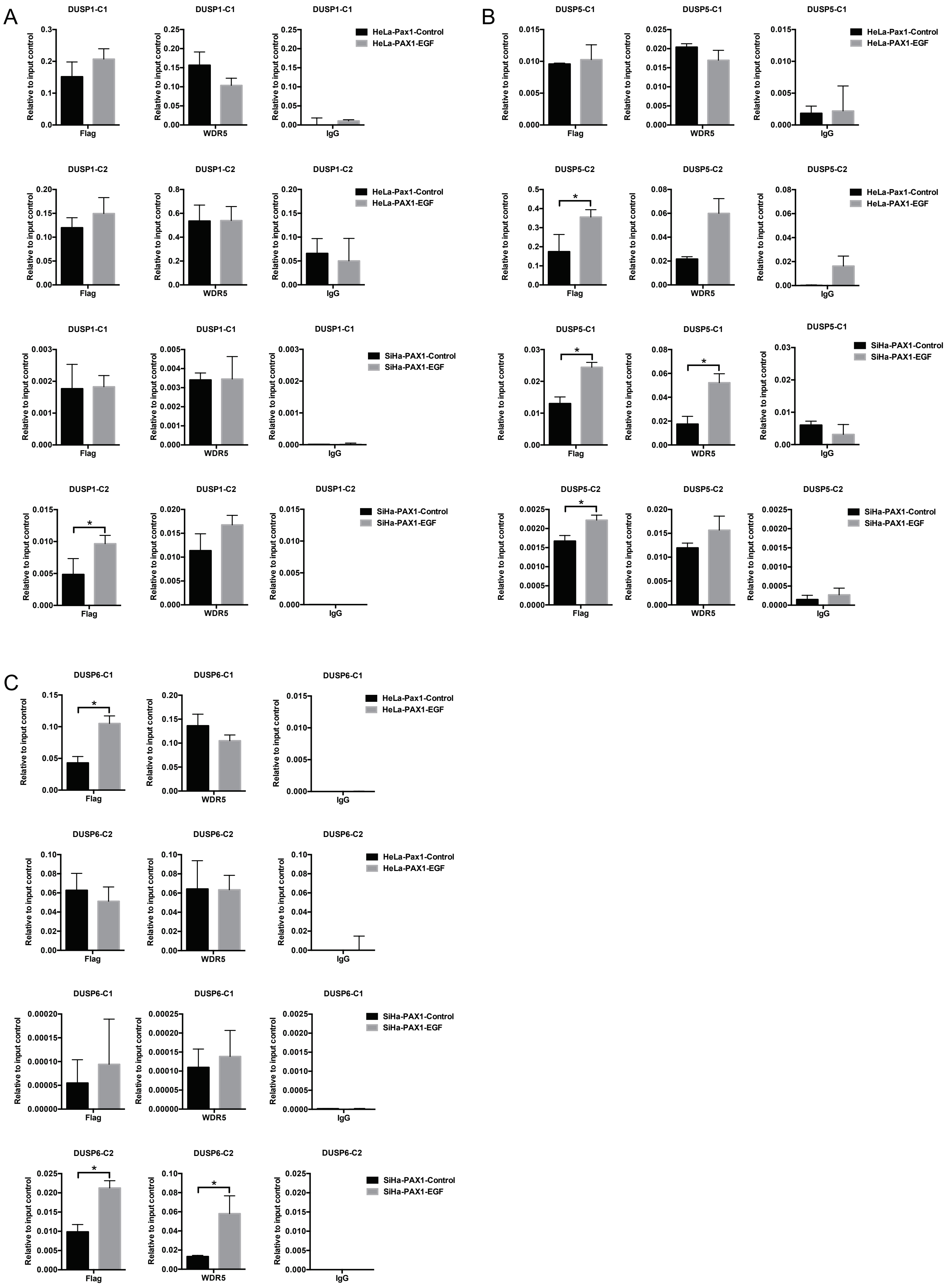

Supplemental Figure 8

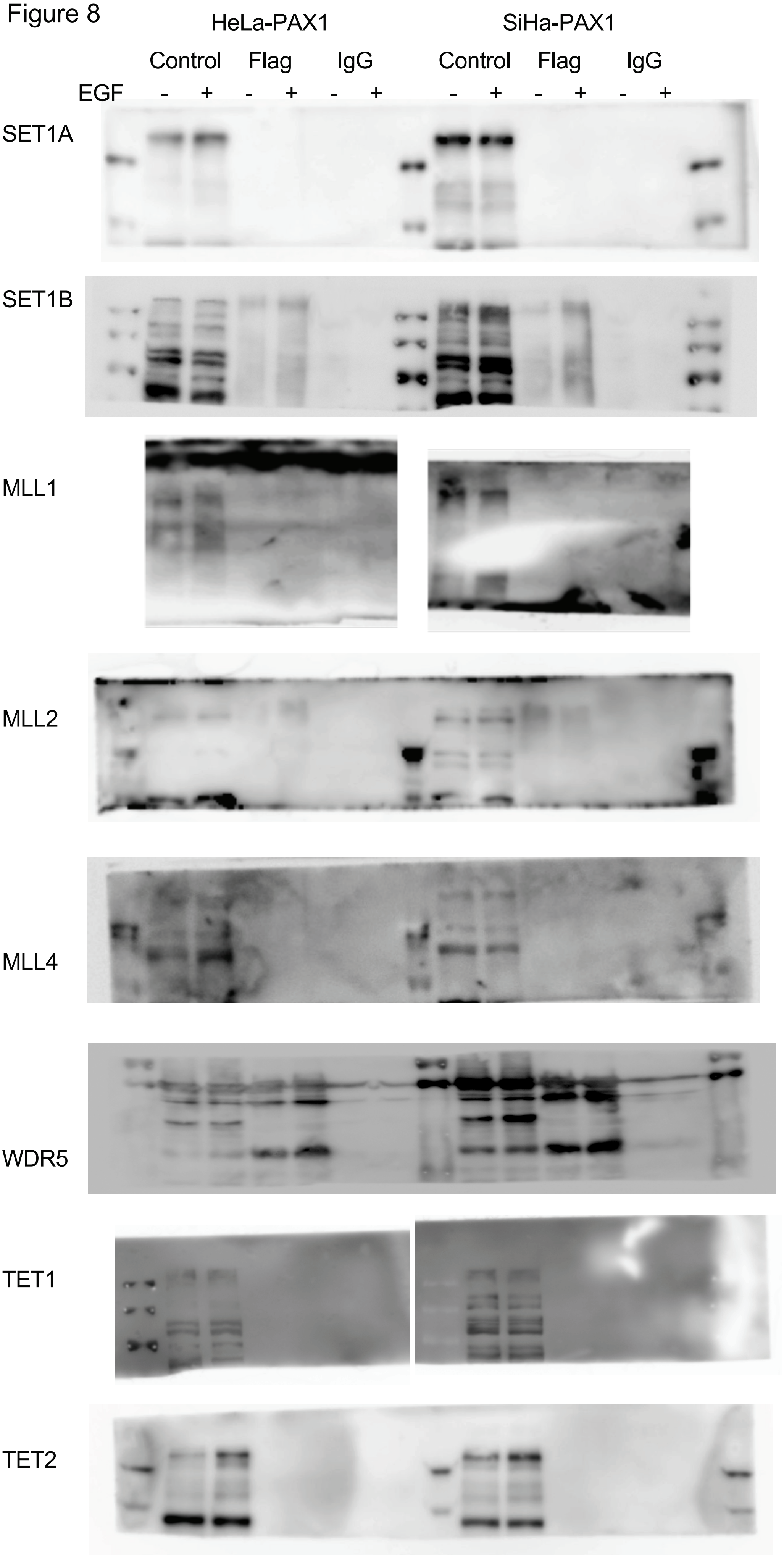

Supplemental Figure 9

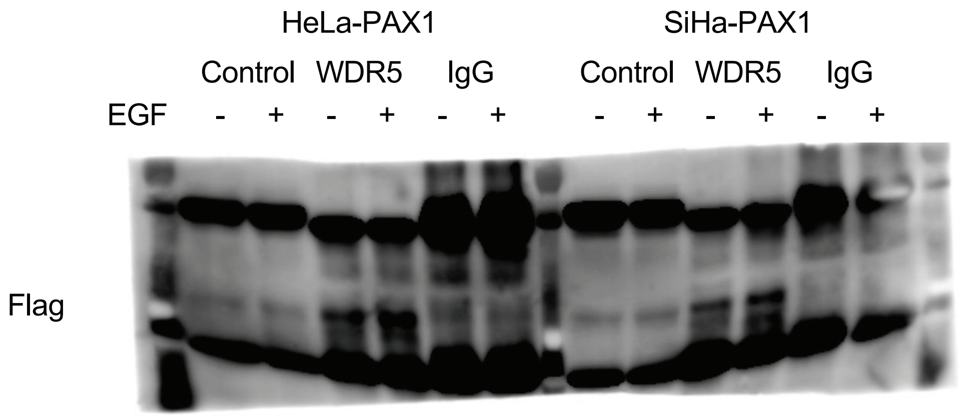

Supplementary figure legends

Supplementary Figure S1. The effect of PAX1 on EMT markers.

(A) HeLa-PAX1 cell. (B) SiHa-PAX1 cell. The expression of EMT marker, SNAIL was downregulated by PAX1 in both cell lines.

Supplementary Figure S2. The quantitative results of PAX1 inhibited kinase pathway.

(A) EGF treatment. (B) IL-6 treatmen.

Supplementary Figure S3. Uncropped blot of Figure 3D.

(**A**) P-p44/42 MAPK. (**B**) p44/42 MAPK.

Supplementary Figure S4. PAX1 inhibited ERK1/2 phosphorylation may through activate PTPRR expression.

(A) RT-qPCR shown the re-expression of PTPRR mRNA in PAX1-overexpressed HeLa and SiHa cells under EGF treatment. (B) ChIP-PCR results of PAX1 bind on PTPRR promoter under EGF treatment.

Supplementary Figure S5. Bioinformatics analysis of PAX1 regulated genes.

(A) Condition of cell for transcriptome analysis. HeLa cell with doxycycline-inducible-PAX1 was treated with EGF for 1 week then treated with EGF and

doxycycline for 2 weeks. Sample were collected before doxycycline treatment, 1-week and 2-week doxycycline treatment. (B) Flowchart of bioinformatics analysis.

Supplementary Figure S6. The binding of PAX1 and WDR5 on Phosphatases' promoter in PAX1 expressed cells after EGF treatment.

(A) DUSP1. (B) DUSP5. (C) DUSP6. PAX1 and WDR5 were confirmed to bind the DUSP1, DUSP5, and DUSP6 promoters. The binding was increased after EGF treatment.

Supplementary Figure S7. The status of histone H3K4 and H3K9 trimethylation on DUSPs promoter in PAX1 expressed cells after EGF treatment.

(A) DUSP1. (B) DUSP5. (C) DUSP6. The H3K4 trimethylation was increased in the promoters of DUSP1, DUSP5, and DUSP6 lacking in changes with H3K9 trimethylation.

Supplementary Figure S8. Uncropped blot of Figure 5E.

Supplementary Figure S9. Uncropped blot of Figure 5F.

Supplementary tables and legends

Symbol	Accession	Symbol	Accession
ACYP2	NM_138448.2	PDP2	NM_020786.1
ALPP	NM_001632.3	PDPR	XM_001134215.1
CTDSPL	NM_005808.2	PPA2	NM_006903.4
DUSP1	NM_004417.2	PPEF1	NM_152224.1
DUSP19	NM_080876.2	PPM1B	NM_001033556.1
DUSP3	NM_004090.2	PPM1K	NM_152542.2
DUSP4	NM_057158.2	PPP1R10	NM_002714.2
DUSP5	NM_004419.3	PPP1R3C	NM_005398.4
DUSP5P	NR_002834.1	PPP2R5A	NM_006243.2
DUSP6	NM_022652.2	PTP4A2	XM_944907.1
DUSP8	NM_004420.2	PTP4A2	XM_944915.1
DUT	NM_001025248.1	PTPLA	NM_014241.3
DUT	NM_001025249.1	PTPLAD2	NM_001010915.1
FLJ30092	XM_933560.1	PTPN20	XM_937744.1
G6PC3	NM_138387.2	PTPN4	NM_002830.2
IMPA2	NM_014214.1	PTPRK	NM_002844.2

Supplementary Table S1. Phosphatases activated by PAX1.

Supplementary Table S2. Pathways affected by PAX1.

Quantitative real-time PCR primers		
Gene	Forward primer	Reverse primer
GAPDH	ACCCACTCCTCCACCTTTGACG	TCTCTTCCTCTTGTGCTCTTG
PAX1	CCTACGCTGCCCTACAACCACATC	TCACGCCGGCCCAGTCTTCCATCT
PTPRR	CATGCTGGATGTAGAAGAAGACA	AACACCCTGTTCTACCTATTCCTG
DUSP1	TTCAACGAGGCCATTGACTT	CCTGGCAGTGGACAAACAC
DUSP4v1	GACATCTGCCTGCTCAAAGG	CAAGGGCTCTGTGGCACT
DUSP4v2	AGCCAGACCGATTCGAGA	AGCAATGGTGGGGCTAGAG
DUSP5	ACAAATGGATCCCTGTGGAA	CCTCCCTTTTCCCTGACAC
DUSP6v1	CGACTGGAACGAGAATACGG	TTGGAACTTACTGAAGCCACCT
DUSP6v2	ACACAGTGGTGCTCTACGAC	CGGGCTTCATCTTCCAGGTA
DUSP8	GACCATTGCGGAGCTCAT	TCATAGACCACCACGTCCTGT
DUSP19v1	GGTTGCTCCTAGGGTCACAA	AGGATGTTGGTTTCAGGCAGA
DUSP19v2	ATGTGCAGGACCTTAGCTCG	AAGAACCACTCCATCCTTATTCTT

Supplementary Table S3. Quantitative real-time PCR primers.

qMS-PCR primers		
Gene	Forward primer	Reverse primer
PTPRR-M1	TTAAGTCGTCGTTAAGATTCGGAGAAG	CCCAACGCCGACCCTCAC
PTPRR-M2	AAGTTGGTGTTGGTTTTTGTCGGC	ACACAACGCAAAAAAACAAACTACTCTCC
DUSP1-M1	GTTGTGGTCGGTTTTTGTTTCG	AAAAACCGCCCGACACG
DUSP1-M2	GATTTAGTTTCGAGGTTGATGACG	CGCAACGAACCTAACCCG
DUSP5-M1	AAGTTTGGTTTACGTGGGTGTTTCGC	ACGCGCGAAACGAATACCGAC
DUSP5-M2	AAGTTTGGTTTACGTGGGTGTTTCGC	CAAATACATCCACCCAAAAACGCTATCCTC
DUSP6-M1	TTTTCGCGGTGTTTACGGTAGGC	AAACCGCGCTTTATCCCACC
DUSP6-M2	AGGGGGGTTTCGGTAGGTATTC	GAAAAACACCAACCGCGCCTAC

Supplementaltry Table S4. qMS-PCR primers.

ChIP-PCR primers		
Gene	Forward primer	Reverse primer
PTPRR-C1	AAGCTGGTGCTGGTTTCTGT	TGCTCTCCGCATAGTGTTTG
PTPRR-C2	GAGCCTACTCTGCTCCAAGC	CAGTAGGAGGTTGCGGGTAA
DUSP1-C1	GCTCGAGTCGGTCTTGGTAG	ACTCTTGTGCCCCTTTTCCT
DUSP1-C2	CCCAGAGGCCGCATATAAAC	CGCTTTTGGACTGAGAGAGG
DUSP5-C1	GTCCTGGTGTCTGATTTGATTG	ATTGTTAGCCTGGCTGGGACT
DUSP5-C2	TCTCCGACGTCCTCTCCTTA	GATCCGGCCTTCAGCTTC
DUSP6-C1	AGGTCCCAATGACTGAAACG	AGGCCTAGGTTGCCAATTTT

Supplementary Table S5. ChIP-PCR primers.
